# The Role of TEMPO/NaBr/NaClO in Hemp Fiber Oxidation: Deciphering the Mechanism and Reaction Kinetics

**DOI:** 10.3390/polym17192629

**Published:** 2025-09-28

**Authors:** Lingping Kong, Peiyu Du, Lizhou Pei, Dan Sun

**Affiliations:** 1 College of Light Industry and Textile, Qiqihar University, Qiqihar 161000, China; 2 Engineering Research Center for Hemp and Product in Cold Region of Ministry of Education, Qiqihar University, Qiqihar 161006, China; 3 Qiqihar Ecological Environment Monitoring Center, Qiqihar 161006, China

**Keywords:** TEMPO oxidation system, industrial hemp staple fibers, carboxyl groups, molecular dynamics, Kinetics, activation energy

## Abstract

In this study, the oxidation of industrial hemp staple fibers by the TEMPO/NaBr/NaClO system was explored by the real-time monitoring of the changes in reaction rate, selective oxidative conversion, and reaction time under different operating conditions such as TEMPO usage, NaBr usage, NaClO usage, reaction time, and reaction temperature. We propose a variable-speed competition mechanism between NaClO and TEMPO, which provides experimental support for the long-standing hypothesis that hypochlorite delays acid formation through modulation of the HOCl/OCl^−^ and HOBr/OBr^−^ equilibrium dynamics. The innovative use of combined analysis for several consecutive first-order reactions to investigate the rate-limiting reactions of TEMPO, TEMPO^+^, and TEMPOH over a range of concentrations revealed that the reaction that generates TEMPOH is the key rate-limiting reaction. We characterize the apparent oxidation kinetics of industrial hemp staple fiber in the TEMPO/NaBr/NaClO system using a pseudo-first-order kinetic model, revealing distinct apparent reaction rates across both primary and secondary bast fiber regions. This paper explained the difference in reaction rate between the two aspects of microfibril spatial structure and cellulose crystal structure. The single-factor analysis indicates that reaction time and temperature exert the most significant influence on the conversion rate of selective oxidation within this system.

## 1. Introduction

Industrial hemp is a cost-effective natural bast fiber with high yields and a long history of cultivation. With the increasing emphasis on the use of renewable biomass to manufacture materials, hemp fiber has received renewed attention and research as an ecological textile raw material [1]. However, a considerable portion of hemp biomass exists in the form of processing residues, especially industrial hemp short fibers produced during mechanical carding. This material is inexpensive and abundant in reserves, but it has not yet been fully utilized. The full development and utilization of these residues through value-added modification is crucial for maximizing resource efficiency. The selective oxidation of cellulose offers a promising route to valorize hemp fiber. By introducing functional groups such as carboxylates, this chemical modification significantly enhances the properties of hemp fibers, unlocking their potential for advanced material applications. The TEMPO/NaBr/NaClO system has emerged as a predominant method for the selective oxidation of cellulose, enabling efficient conversion of C_6_ primary hydroxyl groups to carboxylates under mild aqueous conditions with high region selectivity [2,3,4,5]. Pioneering work by de Nooy et al. (1995) established the foundational mechanism and kinetics for oxidizing water-soluble glucans [6,7]. Subsequent research has extensively applied and optimized this system for various celluloses [8,9]. Kinetic studies, such as those on regenerated cellulose [10] and cotton fibers [11], have typically focused on varying one or two components (e.g., TEMPO or NaBr concentration) and reported simple linear relationships between concentration and reaction rate. While these studies provide valuable insights, a critical gap remains, there is no kinetic study that considers the molecular motion of the three substances (TEMPO, NaBr, NaClO) at the same time. This limitation has constrained the understanding of complex, non-linear kinetic phenomena, such as rate inhibition at higher catalyst concentrations, and has hindered the discovery of unconventional mechanisms within this mature system [12].

**Figure 1:**
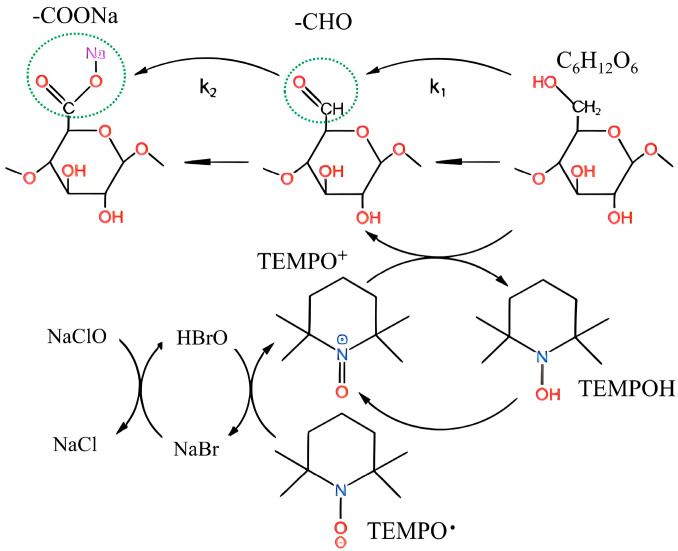
Mechanism of selective oxidation of cellulose by the TEMPO system.

The kinetics were analyzed by monitoring NaOH consumption, and the data were treated with a pseudo-first-order kinetic model to compare the apparent oxidation rates under different conditions, a well-established approach for such heterogeneous systems [10,11]. To validate this hypothesis and decipher the complex interplay within the TEMPO/NaBr/NaClO system, we designed 16 single-factor experiments targeting four critical parameters, namely TEMPO usage, NaBr usage, NaClO usage, and reaction temperature. We systematically investigated the molecular motion dynamics through three quantitative metrics, including oxidation kinetic rate constants, selectivity conversion efficiency, and reaction completion time. Furthermore, a novel joint analysis method for several consecutive first-order reactions was employed to identify the key rate-limiting step among TEMPO-derived species (TEMPO, TEMPO^+^ and TEMPOH). Figure 1 shows a concise relationship of the mechanism, indicating the participation of each substance in the reaction system.

This study experimentally validates the previously proposed hypothesis of the “TEMPO and NaClO variable-speed competition mechanism,” providing a novel explanation for nonlinear kinetics. An innovative analytical approach revealed that the formation of TEMPOH is the key rate-limiting step. A comparative analysis of the kinetics between the primary and secondary bast fiber regions of industrial hemp uncovered correlations between morphological differences and reactivity. Furthermore, changes in the apparent morphology and chemical moieties of industrial hemp staple fibers before and after oxidation were elucidated. This research aimed to decipher the TEMPO-mediated oxidation process of industrial hemp staple fibers, advance molecular dynamics studies, and deepen the understanding of transformation patterns in hemp plant fibers and cellulose oxidation mechanisms. It also offers theoretical support for expanding the range of system substrates, controlling catalytic processes, and developing high-value-added hemp-based biomaterials. The findings of this study contribute to optimizing the oxidation process of hemp fiber residues, enhance the mechanistic understanding of TEMPO-mediated oxidation, and provide insights for future research on multi-step reaction systems and the development of advanced hemp-based materials.

## 2. Materials and Methods

### 2.1. Experimental Materials

Industrial hemp staple fiber (cellulose (52.63%), hemicellulose (19.32%), lignin (18.98%), as well as pectin and ash (9.07%)) was purchased from Qiqihar City, Heilongjiang Province, China; TEMPO was purchased from Alfa Aesar (China) Chemical Co., Ltd. (Shanghai, China); NaOH and NaBr were purchased from Sinopath Chemical Reagent Co., LTD (Shanghai, China); NaClO was purchased from Xilong Science Co., Ltd. (Shantou, China); and KI, H_2_SO_4_, Na_2_S_2_O_3,_ and soluble starch were purchased from Shanghai Titan Technology Co., Ltd. (Shanghai, China).

### 2.2. Experimental Methods

#### 2.2.1. Preparation of Oxidized Industrial Hemp Staple Fibers

Employing a single-factor experimental design, sixteen distinct reaction systems were prepared by combining TEMPO and NaBr at varying concentrations (TEMPO: 0.094, 0.21, 0.38, and 0.78 mmol/L; NaBr: 1.43, 3.24, 5.83, and 11.79 mmol/L) in 750 mL of deionized water. The pH of each resulting TEMPO/NaBr mixed solution was adjusted to 10.0 ± 0.1 by titration with 0.01 mol/L NaOH. Subsequently, 10 g of industrial hemp staple fibers was immersed into each pH-adjusted solution, followed by the addition of a NaClO solution (7% active chlorine) at concentrations of 29.55, 59.11, 109.26, or 236.43 mmol/L, and thoroughly stirred to ensure the rapid and uniform mixing of the oxidant.

Throughout the oxidation process, the pH of the reaction system was maintained at 10.0 ± 0.1 via dynamic titration. Real-time monitoring was performed using a calibrated precision pH meter (Adonis, Dalong Xingchuang Laboratory Instruments (Beijing, China)). Upon the pH dropping to 9.9, a 0.01 mol/L NaOH solution was immediately added in increments (0.625 mL per addition) using a calibrated pipette until pH recovered to 10.1. The operator concurrently recorded the volume of NaOH added and the corresponding reaction time for subsequent kinetic analysis. The reaction was deemed complete when the pH remained stable (with a change ≤0.05 over fifteen consecutive minutes) and no further NaOH consumption occurred. Anhydrous ethanol was then added to terminate the reaction. All titration operations were performed by the same operator to ensure procedural consistency.

#### 2.2.2. Measurement of Glucuronide Production

**Figure 2:**
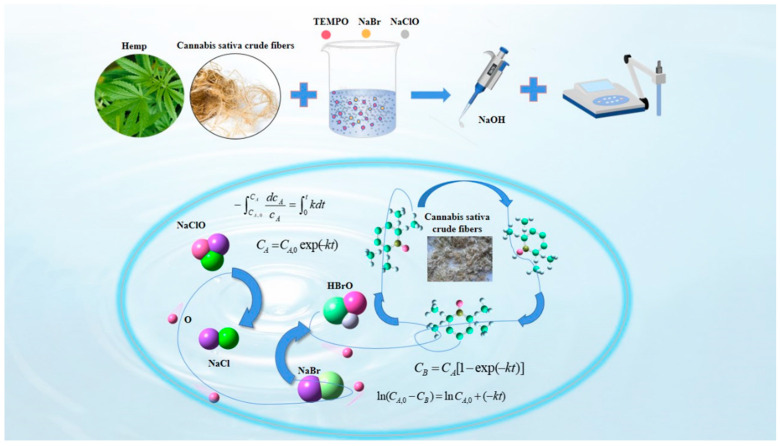
Diagram of the experimental procedure.

We employed in situ physical characterization techniques to quantitatively assess the oxidation kinetics rate during TEMPO-mediated cellulose oxidation, enabling real-time monitoring of reaction progress through spectral and electrochemical parameter tracking. The production of glucuronic acid (mol/L) was monitored indirectly by measuring the consumption of NaOH (mL/min) to find the concentration of the product as a function of time, making a c-t image. The flowchart of the experiment is shown in Figure 2.

Sun et al. concluded from the ^13^CNMR spectra that the difference in NaOH consumption and side reactions was only 8% for the selective oxidation of regenerated cellulose by the TEMPO/NaBr/NaClO system [10]; Isogai et al. oxidized different natural cellulose by NaBH_4_ reduction TEMPO/NaBr/NaClO and NaBr/NaClO, and measured the number of carboxyl groups in cotton and ramie samples after oxidation, and the number of carboxyl groups produced by NaBr/NaClO oxidation was only 4.8% and 7.0% of that produced by TEMPO/NaBr/NaClO oxidation [13]. These studies confirm that under our experimental conditions (pH = 10.0, large excess of NaClO and NaBr), the NaOH consumption predominantly reflects the selective conversion of C6 primary hydroxyl groups to carboxylates. Therefore, titrimetric monitoring of NaOH consumption is a robust analytical tool for elucidating the kinetics of TEMPO-mediated selective oxidation [11,12].

#### 2.2.3. Establishment of Rate Equations for Oxidation Kinetics

The kinetic derivation presented below focuses on the concentration change of the cellulose substrate. The reactions involving the TEMPO catalyst (Equations (6)–(8)) proceed rapidly. Under conditions of excess NaClO/NaBr, the concentration of the active oxidizing species (TEMPO^+^) remains relatively constant, enabling effective catalyst regeneration. Based on these premises, the oxidation of cellulose hydroxyl groups can be treated using a pseudo-first-order kinetic model. (1) Within this framework, the observed apparent rate constant, kapp, inherently embodies the net contribution of these catalytic steps.

Specifically, kapp serves as a macroscopic kinetic parameter that integrates both the intrinsic chemical reaction rate, k, and the influence of physical mass transfer processes (such as reactant diffusion and fiber surface accessibility) inherent to the actual heterogeneous reaction system. To elucidate this relationship more clearly, we introduce the following conceptual equation:

(1) $$k_{app}\approx \eta \times k$$

Here, η (0 < η ≤ 1) represents an efficiency factor, primarily accounting for the rate reduction due to diffusion limitations. Consequently, kapp provides a practical metric for comparing the overall oxidation efficiency under different reaction conditions, while explicitly acknowledging that the intrinsic rate constant k could be higher under ideal, diffusion-unlimited conditions. For the sake of conciseness, kapp will be referred to simply as k in the subsequent sections.

A pseudo-first-order kinetic model was applied to treat the data, based on the premises that the surface reaction is rate-limiting under our conditions and that the aqueous oxidant concentrations (NaClO, NaBr) were in large excess and thus approximately constant. This approach, following field convention [10,11,14], aims to quantify and compare the apparent reaction rates. Based on the mechanism shown in Figure 1, the oxidation of the primary alcohol to the aldehyde by TEMPO^+^ (Equation (9)) is the rate-determining step, as the subsequent oxidation of the hydrated aldehyde (Equation (11)) is much faster [7,10]. The detailed principle is as follows, which can be divided into the following three steps:

(1) Formation of oxidant HOBr.

(2) $$OCl^{-}+H_{2}O⇌HOCl+OH^{-\;\;}$$

(3) $$HOCl+Br^{-}\to HOBr+Cl^{-}$$

(4) $$HOBr+OH^{-}⇌OBr^{-}+H_{2}O$$

(5) $$OCl^{-}+Br^{-}⇌OBr^{-}+Cl^{-}$$

Under the alkaline conditions (pH = 10) described in this study, equilibrium Equation (5) is the dominant pathway for the generation of the active oxidant (OBr^−^).

(2) Formation of primary oxidant TEMPO^+^.

(6) $$(H)OBr+2TEMPO•\to 2TEMPO^{+}+OH^{-}+Br^{-}$$

(7) $$\left(H\right)OBr+TEMPO•\to TEMPO^{+}+Br^{-}+H_{2}O$$

(8) $$TEMPO^{+}+TEMPOH\to 2TEMPO•$$

(3) Formation of cellulosic aldehyde acids.

(9) $$R-CH_{2}OH+TEMPO^{+}+OH^{-}\overset{k1}{\to }\;R-CHO+TEMPOH+H_{2}O$$

(10) $$R-CHO+H_{2}O⇌{\left[R-CHO-H_{2}O\right]}_{\left(hydrated\;form\right)}$$

(11) $$[R-CHO-H_{2}O]+\left(H\right)OCl\overset{k2}{\to }R-COOH+Cl^{-}+2H_{2}O$$

k_1_ and k_2_ are rate constants. Since the oxidation rate of hydrated aldehydes is much faster than that of primary alcohols, the reactions of the TEMPO/NaBr/NaClO oxidation system to oxidize industrial hemp staple fibers can be regarded as a continuous first-order reaction [7,11]. k_2_ > k_1_, so the oxidation of the primary alcohol to the aldehyde (Equation (9)) is the rate-determining step of the reaction.

For a pseudo-first-order reaction, the rate of disappearance of the reactant (C_6_ primary hydroxyl groups, denoted as A) is given by:

(12) $$-\frac{{dc}_{A}}{dt}={kc}_{A}$$

Separating the variables, one gets $-\frac{{dc}_{A}}{c_{A}}=kdt$.

Reaction time from 0→t, and the concentration of the corresponding component A from $c_{A,0}\to c_{A}$

$$-\int_{c_{A,0}}^{c_{A}}\frac{{dc}_{A}}{c_{A}}=\int_{0}^{t}kdt$$

Integral and exp reduction equation:

(13) $$c_{A}=c_{A,0}\exp(-kt)$$

Therefore, the oxidation rate equation for the oxidation of industrial hemp staple fiber by the TEMPO/NaClO/NaBr system is
[CELL-CH_2_OH]_t_ = [CELL-CH_2_OH]_0_ exp(−k_1_t)[CELL-COOH]_t_ = [CELL-CH_2_OH]_0_ {k_1_/k_2_ − k_1_} {exp(−k_1_t) − exp(−k_2_t)}

After simplification and conversion:[CELL-COOH]_t_ = [CELL-CH_2_OH]_0_ {1 − exp(−k_1_t)}Ln([CELL-CH_2_OH]_0_ − [CELL-COOH]_t_) = −k_1_t + ln [CELL-CH_2_OH]_0_
where [CELL-CH_2_OH]_0_ and [CELL-CH_2_OH]_t_ represent the molar concentration of the primary hydroxyl group at the C_6_ position at the onset of the reaction and the molar concentration of the glucuronic acid produced at the reaction time t, respectively (which can be indirectly estimated by the concentration of molar NaOH consumed in the reaction process). [COOH]_t_ denotes the concentration of carboxyl groups formed at time t. For simplicity and uniformity, C[CELL-CH_2_OH]_t_ and C[CELL-CH_2_OH]_0_ are set as C_B_ and C_A,0_ in this paper, and the above equation can be simplified as

(14) $$c_{B}=c_{A,0}[1-\exp(-kt)]$$

(15) $$c_{A,0}-c_{B}=c_{A,0}\exp(-kt)$$

For both sides of the equation, taking the logarithm with e as the base can estimate the value of the rate constant k.

### 2.3. Evaluation Methodology

#### 2.3.1. Scanning Electron Microscope Analysis

The morphological structure of the industrial hemp staple fibers before and after oxidation by the TEMPO system, alongside a control group subjected to identical conditions without the addition of oxidizing agents, was observed using a scanning electron microscope (SEM, JSM-7800 F, HITACHI, Tokyo, Japan), respectively. The test conditions included accelerating voltage, relative humidity, and temperature, set at 5 kV, 65%, and 20 °C. Before testing, vacuum drying and platinum spraying are necessary to improve the observation of the samples.

#### 2.3.2. FT-IR Analysis

The dried sample was crushed and mixed with potassium bromide in a ratio of about 1:150 under infrared light and ground uniformly in an agate mortar. Subsequently, appropriate quantities of the mixture were extracted and test tablets were made using a tablet press. Infrared spectroscopy (FT-IR, Spectrum-One B, Perkin Elmer, Wellesley, MA, USA) with a scanning wavelength range of 4000–400 cm^−1^ was used to investigate the chemical structure of the industrial hemp staple fibers before treatment and the chemical structure of the morphological structure of the optimum group of industrial hemp staple fibers after oxidation by the TEMPO system.

## 3. Results and Discussion

### 3.1. Analysis of Apparent Oxidation Process

**Figure 3:**
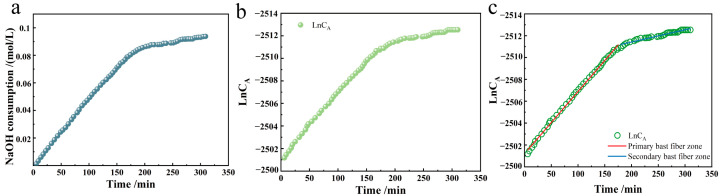
Oxidation kinetics plots ((**a**). NaOH consumption variation curve for TEMPO system mediated oxidation reaction of industrial hemp staple fibers, (**b**). LnC_A_-t reaction rate curve, (**c**). LnC_A_-t reaction rate fitting curve).

We constructed the kinetic consumption profile of NaOH during TEMPO/NaBr/NaClO-mediated selective oxidation of industrial hemp staple fibers, with real-time titration data presented as cumulative NaOH consumption (mmol) versus reaction time (min) in Figure 3a. The experimental conditions were as follows: 750 mL H_2_O, 0.21 mmol/L TEMPO, 3.24 mmol/L NaBr, 109.26 mmol/L NaClO (7.0% effective chlorine content), pH 10, temperature 20 °C, reaction time 5 h.

The Ln-t image and its reaction rate graph were made according to the primary reaction rate equation, as shown in Figure 3b,c.

From the reaction rate curve, it can be seen that the chemical reaction rate remained relatively stable at the reaction time of 0–180 min. The reaction rate slightly decreased after 180 min, but the chemical reaction continued. This result aligns with the data on the oxidation of other natural celluloses using the TEMPO/NaBr/NaClO method [14]. The structural characterization of industrial hemp fibers reveals that the stem bast fibers are primarily localized in the middle and outer cortical layers, exhibiting a porous microstructure with loose fibrillar packing. This anatomical feature facilitates reagent penetration during TEMPO-mediated oxidation. The primary bast fiber has higher secondary wall thickness and cell wall thickness than the secondary bast fiber, which accounts for about 92–95% of the fiber composition [15,16,17]. In contrast, secondary bast fibers lie closer to the pith, where the junction region reaction struggles to proceed, resulting in a lamellar spatial structure. The microfiber angle of the S1 layer of hemp varies between 70–90°, the microfiber angle of the outer layer of the S2 layer varies between 25–30°, and the microfiber angle of the inner layer of the S2 layer varies around 0–5° [18]. Carrying out the oxidation reaction becomes challenging due to the significant variation in the angle of arrangement of the microfibers in each layer. In this paper, we refer to the initial reaction period as the oxidation of primary bast fibers and the later stages as the oxidation of cannabinoid secondary bast fibers. In addition, xylan in hemp fibers was mainly concentrated in the primary wall of the thick-walled bast tissue, differing from its distribution in the secondary wall of other dicotyledonous plants (where galactans primarily accumulated in thin-walled cells) [17]. This suggests that hemicellulose participated in both reaction segments [19,20]. Most of them degrade into water-soluble substances [21]. Lignin in hemp fibers primarily accumulates in the intercellular layer and primary wall [18], and its removal depends on oxidant concentration and oxidation time.

The reaction rate curves for the oxidation of industrial hemp staple fibers by the TEMPO system shown in Figure 3 above were fitted to obtain the corresponding reaction rate constants and fitting coefficients, the values of which are shown in Table 1.

The coefficient of determination R^2^ removes the effect of the degree of dispersion of the raw data, so the high coefficients of determination of the primary and secondary bast fibers demonstrate that the reaction in both regions conforms to the first-order kinetic equations, with rate constants of k_1_ = 1.17 min^−1^ in the primary bast region and k_2_ = 0.18 min^−1^ in the secondary bast region. By analyzing the reaction rate constants of these two curves, it can be seen that the reaction rate of the TEMPO/NaBr/NaClO system for oxidation of the primary bast zone of industrial hemp staple fibers is 6.5 times higher than that of the secondary bast zone. This difference in reaction rates highlights the different reactivity and kinetics of the oxidation process of hemp primary and secondary bast fibers.

The observed plateau in the oxidation kinetics and the attainable degree of conversion are not a limitation of the process, but rather a direct manifestation of the intrinsic structural heterogeneity of industrial hemp staple fibers. This heterogeneous material comprises both readily oxidizable primary bast fibers and more recalcitrant secondary bast fibers. The applied mild oxidation conditions are strategically designed to selectively functionalize the accessible domains while preserving the integrity of the cellulose polymer chains. The successful application of kinetic deconvolution to model these distinct reaction phases provides a valuable framework for assessing and exploiting the differential reactivity within such complex lignocellulosic feedstocks.

The application of the pseudo-first-order model is predicated on the assumption that the oxidation of the aldehyde intermediate (Equation (11)) is significantly faster than the initial oxidation of the alcohol to the aldehyde (Equation (9)), i.e., k2≫k1 [11,13]. This foundational assumption, widely supported in previous studies of the TEMPO/NaBr/NaClO system [6,10,11] ensures that the aldehyde concentration remains negligible throughout the reaction.

### 3.2. Effect of TEMPO Usage

**Figure 4:**
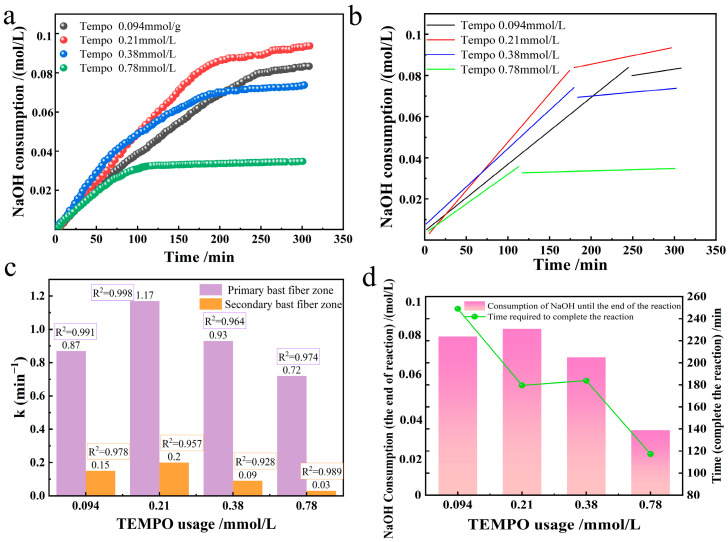
Effect of TEMPO usage on the oxidation of industrial hemp staple fibers ((**a**). NaOH consumption; (**b**). its linear fitting; (**c**). reaction rate constant graph; (**d**). plot of reaction end time and NaOH consumption at reaction end).

In the TEMPO system, the amount of TEMPO was varied to observe the effect on the oxidation system, selectively oxidizing industrial hemp staple fibers produced. Under the alkaline environment of room temperature 20 °C and pH = 10, 3.24 mmol/L NaBr, 109.26 mmol/L NaClO, and 10 g industrial hemp staple fibers were dissolved in 750 mL deionized water. We tested four TEMPO usage groups (0.094, 0.21, 0.38, 0.78 mmol/L), corresponding to solution concentrations of 0.0015%, 0.003%, 0.006%, and 0.012%, to compare changes in reaction rate constants. We measured NaOH consumption during the preparation of oxidized industrial hemp staple fibers across these TEMPO usages, performed linear fitting, and present the results in Figure 4.

The usage of TEMPO catalyst has a profound non-monotonic effect on the oxidation kinetics of industrial hemp fibers, which was quantified by the apparent first-order rate constant k. As shown in Figure 4c, the k of the primary phloem fibers exhibits a distinct volcanic trend. With the increase of TEMPO concentration, k first rises sharply and then drops sharply. The observed initial positive correlation (0–0.094 mmol/L TEMPO) is consistent with the classical catalytic behavior. Increasing the catalyst load will enhance the steady-state concentration of the active oxidant (TEMPO^+^), thereby accelerating the rate-determining step: the oxidation of primary alcohols to aldehydes Equation (8). The increase in the proportion of cumulative NaOH consumption at the completion of the reaction confirmed this (a dual-axis plot Figure 4d), indicating an improvement in conversion efficiency within this dose range.

However, the decrease in k after exceeding the optimal dose (0.21–0.78 mmol/L TEMPO) indicates that there are more complex interactions in the reaction system. This reverse relationship indicates that an excessive TEMPO begins to inhibit the process it was supposed to catalyze. We believe that this inhibition stems from the variable-speed competition mechanism, which is at the core of our hypothesis. Excessively high TEMPO may disrupt the critical equilibrium between catalytic substances (TEMPO/TEMPO^+^/TEMPOH). Specifically, high [TEMPO] can transfer the equilibrium of the regeneration reaction (Equation (7), TEMPO + TEMPO + 2TEMPO•) to the reactants, effectively isolating TEMPO^+^ and hindering its efficient oxidation of cellulose substrates. This creates a competitive environment in which TEMPO molecules interfere with each other, ultimately suppressing the overall catalytic throughput.

Three-dimensional comprehensive analysis shows that in the TEMPO variable group, the trend of oxidation reaction rate constant and NaOH consumption at the end of the reaction is consistent. Under the premise that the concentration of BrO^−^ remained unchanged, with the increase of TEMPO concentration, the oxidation reaction rate and the generation of glucuronic acid increased, and the reaction time became shorter, indicating that the reaction rate of aldehyde-related k_1_ accelerated. With the acceleration of reaction rate k_1_, which means that the generation rate of BrO^−^ accelerates, NaClO becomes less and less in the reaction, and the time required to the end of the reaction is related to the consumption rate of NaClO. TEMPO is competitive with polysaccharides against secondary oxides, and a shorter reaction time can reduce the damage to fibers and slightly increase the selective oxidation yield [22,23]. When the TEMPO usage increased from 0.094 mmol/L to 0.21 mmol/L, the reaction rate k_1_ of the aldehyde group increased by about a quarter. The consumption of TEMPO^+^ is divided into two routes: the oxidation kinetic rate equation building part Equation (8)/Equation (9). In Equation (8), TEMPO^+^ reacts with TEMPOH to form 2 TEMPO•, which corresponds to Equation (6)/Equation (7) to form 2 TEMPO^+^, one of which continues back into the reaction cycle in Equation (8). The reaction of TEMPO^+^ with TEMPOH produces only one effective TEMPO• involved in the subsequent reaction. In Equation (9), TEMPO^+^ is converted to TEMPOH by reaction with C_6_-OH. According to the established mechanism [7,8,9], the order of occurrence of these two reactions is first the reaction with the cellulose hydroxyl (Equation (9)) to generate TEMPOH, followed by the disproportionation reaction (Equation (8)). The rate of reaction Equation (9) is exactly increased by about 50% when the amount of TEMPO is doubled, these results indicate that the formation of aldehyde groups by C_6_-OH in oxidized cellulose is the key rate-limiting reaction in a series of TEMPO-related reactions.

Notably, the observed near-linear increase in carboxyl group formation (Figure 4b) during the initial phase at optimal TEMPO usage (0.21 mmol/L) deviates from a perfect first-order decay curve. This apparent zero-order kinetic characteristic suggests that within this specific concentration window, the rate of aldehyde formation is not limited by the concentration of cellulose hydroxyls, but rather by the steady-state concentration of the active oxidant (TEMPO^+^), which is maintained by the dynamic balance between its generation (from NaClO/NaBr) and consumption (reduction to TEMPOH). This observed plateau in rate provides strong experimental support for the proposed ’variable-speed competition mechanism’ between TEMPO and NaClO, wherein an optimal equilibrium exists. When the amount of TEMPO was greater than 0.21 mmol/L, the rate constant of the oxidation reaction and the amount of NaOH consumed to the end of the reaction did not increase but decreased, therefore, when the amount of TEMPO was greater than 0.21 mmol/L, the selective oxidation reaction would be inhibited by an excessive amount of TEMPO. The rate constant of the reaction was the smallest when the TEMPO usage of 0.78 mmol/L was used, the time taken to the end of the reaction was the shortest, and the least amount of glucuronic acid was produced. Excess TEMPO hinders the conversion of TEMPOH, resulting in a slowing of the reaction rate k_1_ for the generation of aldehyde groups. At this time, the consumption rate of OCl^−^ is faster than that under the condition that TEMPO is less than 0.21 mmol/L, and the reaction rate k_2_ of carboxyl group formation is slowed down with the formation of more Cl^−^.

Researchers must account for the number of independent variables when comparing multiple regression equations with differing predictor counts. During the experiment, the measurement period of the instrument used impacted the sample measurement interval, resulting in a different number of samples in each group of data. We use coefficient-corrected measurements as final results to ensure experimental accuracy. As can be seen from the data in Figure 4c, the primary and secondary bast fibers have high coefficients of determination R^2^, suggesting that the response of these two regions at different TEMPO usages is consistent with the first-order kinetic equation.

### 3.3. Effect of NaBr Usage

**Figure 5:**
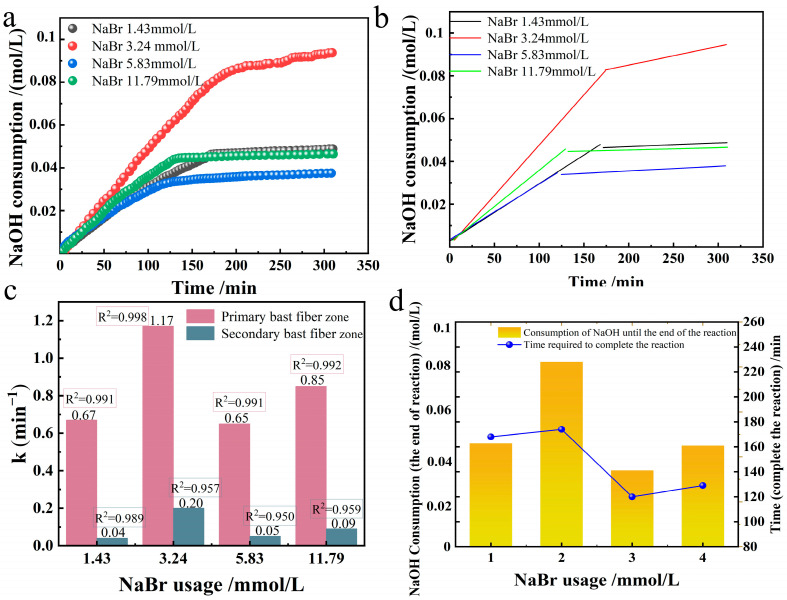
Effect of NaBr usage on the oxidation of industrial hemp staple fibers ((**a**). NaOH consumption; (**b**). its linear fitting; (**c**). reaction rate constant graph; (**d**). plot of reaction end time and NaOH consumption at reaction end).

We investigated how NaBr usage affects the selective oxidation of industrial hemp fibers in the TEMPO/NaBr/NaClO system. In an alkaline environment at room temperature 20 °C and pH = 10, 0.21 mmol/L of TEMPO, 109.26 mmol/L of NaClO, and 10 g of industrial hemp staple fiber were taken and dissolved in 750 mL of deionized water. Four groups of different NaBr usages (1.43, 3.24, 5.83, 11.79 mmol/L) corresponding to solution gradient configurations (0.015%, 0.03%, 0.06%, 0.12%) were set, respectively, to compare the change of reaction rate constant. We plotted NaOH consumption for oxidized hemp fibers across different NaBr usages and performed linear fitting, as shown in Figure 5.

The relationship between the reaction rate constants of primary and secondary bast fibers for the preparation of oxidized industrial hemp staple fibers at different NaBr usages is shown in Figure 5c. When the amount of NaBr is within the range of (0, 3.24], there is a positive correlation between the amount of NaBr and the reaction rate constant of oxidation. The larger the amount of NaBr, the larger the oxidation reaction rate constant. The peak value of the oxidation reaction rate constant is consistent with the data of the oxidized viscose fibers in this system [10]. The NaBr usage was negatively correlated with the reaction rate constant of oxidation when the NaBr usage was in the range of [3.24, 5.83], and the larger the NaBr usage, the more diminutive the oxidation reaction rate constant, and this proportionality was in agreement with the results of the study of oxidation of cotton fibers by this system [11]. When the usage of NaBr was within the range of [5.83, 11.79], the reaction rate constant increased slightly with the increase of the usage of NaBr, which was observed for the first time.

A two-dimensional plot simultaneously showing reaction time and NaOH consumption was drawn, as shown in Figure 5d. When the NaBr usage was in the range of (0, 3.24], there was a positive correlation between the NaBr usage and the amount of NaOH consumed up to the end of the reaction, i.e., the more NaBr usage was used, the more glucuronic acid was produced, and the higher the selective oxidative conversion rate was; When the amount of NaBr was within the range of [3.24, 5.83], the amount of NaBr was negatively correlated with the consumption of NaOH to the end of the reaction, that is, the more NaBr was used, the less glucuronic acid was generated, and the lower the conversion rate of selective oxidation was. When the NaBr usage was in the range of [5.83, 11.79], there was a slight recovery in NaOH consumption to the end of the reaction. Therefore, when the usage of NaBr is 3.24 mmol/L, the selective oxidation conversion rate is the highest, and the selective oxidation conversion rate increases nearly twice as much as when the usage of NaBr is 1.43 mmol/L. When the usage of NaBr is 11.79 mmol/L, the selective oxidation conversion is similar to that when the usage of NaBr is 1.43 mmol/L. From the dimension of time required until the end of the reaction, the time needed for the NaBr usage of 1.43 mmol/L and 3.24 mmol/L groups to the end of the reaction is around 170 min. The amount of NaBr used was 5.83 mmol/L and 11.79 mmol/L, and the time required for the reaction to end was about 130 min for both groups. However, when the usage of NaBr was between 3.24 mmol/L and 5.83 mmol/L, the time to the end of the reaction was significantly decreased by 23.5%.

In the three-dimensional analysis, the trends of the oxidation reaction rate constants, the amount of NaOH consumed until the end of the reaction, and the time required until the end of the reaction under different NaBr usage conditions were in the same direction. In 1995, De Nooy observed that hypochlorite delayed acid formation, presumably due to the influence of HOCI/OCI^−^ and HOBr/OBr^−^ equilibria [6]. This experiment shows that there is a dynamic equilibrium between OCI^−^ and BrO^−^. Since the concentration of BrO^−^ and OCI^−^ in equilibrium depends on the concentration base of Br^−^, the oxidation rate of OCl^−^ to Br^−^ is much higher than the reduction rate of OBr^−^ when the amount of NaBr is small, and the reaction rate also increases with the increase of NaBr. OCl^−^ and OBr^−^ concentrations tend to the equilibrium maximum concentrations. During this period, NaClO, which did not participate in the reaction, accelerated the rate of non-selective oxidation and depolymerization, resulting in less glucuronic acid being produced [22,23]. When the optimal value of NaBr is 3.24 mmol/L, it oxidizes rapidly with NaClO, and the cellulose is less damaged, which lays the foundation for long-term, high-rate, and highly selective oxidation conversion. The time required to the end of the reaction was similar for the first two groups, consistent with the conclusion that NaClO usage correlates with reaction time in the TEMPO group analysis. When NaBr is greater than 3.24 mmol/L, the concentration of BrO^−^ is equal to or greater than the concentration of OCl^−^. After the reaction of Br^−^ with HClO, the excess remaining Br^−^ as a product will hinder the conversion of TEMPO, inhibiting the reaction rate k_1_ for the formation of aldehyde groups. At the same time, a large amount of Br^−^ is converted to BrO^−^ by HOCl and accompanied by the production of Cl^−^, which leads to a decrease in reactants and an increase in products in reactions that generate carboxyl groups. The k_1_ and k_2_ reactions, which are directly related to the formation of uronic acid, are simultaneously hindered. This reduction in the oxidation rate constant, the consumption of NaOH, and the reaction time to completion is very unfavorable for the reaction.

According to the method “Effects of different TEMPO usage on oxidation rate constant, selective oxidation rate and time to the end of reaction”, the effects of different NaBr usages on oxidation rate constant, selective oxidation conversion rate, and time to the end of reaction were analyzed, the high coefficients of determination for primary and secondary bast fibers indicate that the responses of different NaBr usage groups to both regions are by the first-order kinetic equation.

### 3.4. Effect of NaClO Usage

**Figure 6:**
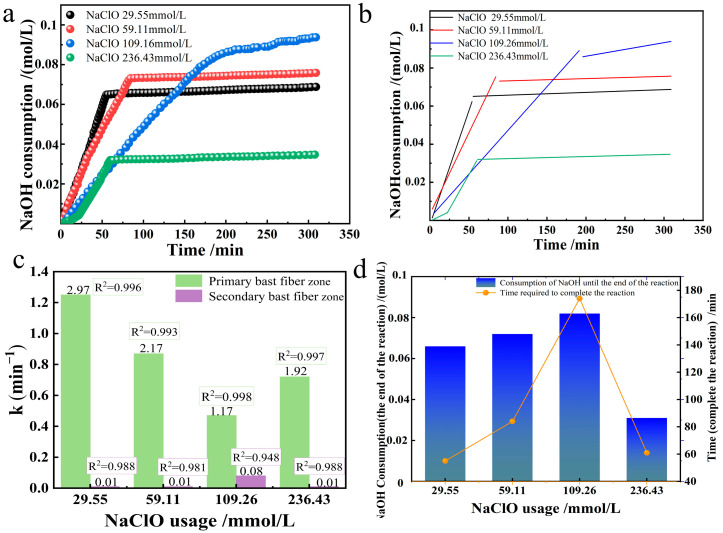
Effect of NaClO usage on the oxidation of industrial hemp staple fibers ((**a**). NaOH consumption; (**b**). its linear fitting; (**c**). reaction rate constant graph; (**d**). plot of reaction end time and NaOH consumption at reaction end).

The effect of selective oxidation of industrial hemp staple fibers produced by the oxidation system was observed by varying the amount of NaClO in the TEMPO/NaBr/NaClO system. Under the alkaline environment of room temperature 20 °C and pH = 10, 0.21 mmol/L TEMPO, 3.24 mmol/L NaBr, and 10 g industrial hemp staple fibers were dissolved in 750 mL deionized water, four groups of different NaClO usages (29.55, 59.11, 109.26, 236.43 mmol/L) corresponding to the solution gradient ratio configurations (0.15%, 0.3%, 0.6%, 1.2%) were added to observe the changes in the oxidation reaction rate constant, as shown in Figure 6c. The NaOH consumption of oxidized industrial hemp staple fibers at different NaClO usages and their linear fitting plots were plotted as shown in Figure 6a,b.

The relationship between the reaction rate constants of primary and secondary bast fibers when oxidizing industrial hemp staple fibers at different NaClO usages is shown in Figure 6c. The amount of NaClO in the primary bast fibers was negatively correlated with the oxidation rate constant when the NaClO usage was in the range of (0, 109.26], and the larger the NaClO usage, the smaller the oxidation rate constant; in the range of [109.26, 236.43], the reaction rate constant increased slightly with the increase in the amount of NaClO.

A two-dimensional plot simultaneously showing reaction time and NaOH consumption was drawn, as shown in Figure 6d. When the amount of NaClO is within the range of (0, 109.26], the concentration of NaClO is positively correlated with the consumption of NaOH to the end of the reaction; that is, the more the amount of NaClO, the more glucuronic acid will be generated, and the higher the selective oxidation conversion rate will be. This rule is used as a process regulation in the preparation of cellulose nanocrystals [19]. At the NaClO usage of [109.26, 236.43], the NaClO concentration was negatively correlated with the amount of NaOH consumed up to the end of the reaction, i.e., the higher the NaClO usage, the less glucuronic acid was produced, and the lower the selective oxidative conversion rate. At the amount of NaClO of 236.43 mmol/L, the initial slope of the first linear fit is relatively low, and then turned up, probably due to the large amount of NaClO added, causing the solution pH to be too high and inhibiting the reaction. From the dimension of time required to the end of the reaction, when the amount of NaClO is within the range of (0, 109.26], the amount of NaClO is positively correlated with the time required to the end of the reaction; that is, the more NaClO is used, the longer the reaction time will be. When the amount of NaClO was 109.26 mmol/L, the time required to the end of the reaction was 174 min, after which the amount of NaClO was increased to 236.43 mmol/L g, and the time required to the end of the reaction was shortened, which was close to the time required to the end of the reaction for the amounts of NaClO 29.55 and 59.11 mmol/L.

Three-dimensional comprehensive analysis shows that when the amount of NaClO is (0, 109.26], the higher the total amount of NaClO, the higher the selective conversion rate and the higher the consumption of NaOH at the end of the reaction. Since Cl^−^ cannot be converted into additional ClO^−^, this means that the reaction will eventually terminate as the ClO^−^ is consumed, suggesting that the selective oxidation fails to proceed adequately when the amount of NaClO is low. With the increase in the amount of NaClO, the reaction speed gradually decreases. Considering that there are two formation routes of Cl^−^, which is twice that of Br^−^, it means that while the equilibrium concentration of OBr^−^ and OCl^−^ increases, more and more Cl^−^ prevents the conversion of Br^−^ to BrO^−^.

The effects of different NaClO usages on the oxidation rate constants, selective oxidation rates, and time to the end of the reaction were analyzed by referring to the method of “Effects of different TEMPO usages on the oxidation rate constants, selective oxidation rates, and time to the end of the reaction”. The high coefficients of determination for primary and secondary bast fibers indicate that the reactions in these two regions are consistent with the first-order kinetic equation at different NaClO usages.

### 3.5. Effects of Temperature Changes

**Figure 7:**
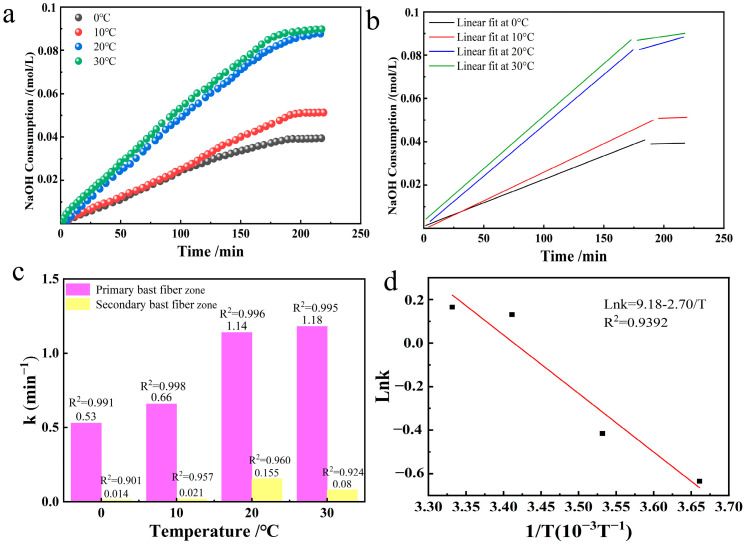
Preparation of oxidized industrial hemp staple fibers at different temperatures ((**a**). NaOH consumption; (**b**). its linear fit; (**c**). reaction rate constant graph; (**d**). Arrhenius plot in the range 0–30 °C).

The effects produced during the selective oxidation of industrial hemp staple fibers were observed by varying the reaction temperature in the TEMPO/NaBr/NaClO system. We took 0.213 mmol/L of TEMPO, 3.24 mmol/L of NaBr, 109.26 mmol/L of NaClO, and 10 g of industrial hemp staple fiber dissolved in 750 mL of deionized water, adjusted the solution pH = 10, and compared the changes in the rate constants of the reaction at the temperatures of 0 °C, 10 °C, 20 °C, and 30 °C. The NaOH consumption in the reaction of oxidized industrial hemp staple fibers at different temperatures and its linear fitting graphs were plotted as shown in Figure 7a,b.

According to the Figure 7c data, the oxidation reaction in the primary bast fibers exhibits a high coefficient of determination, indicating that the reaction in this region complies with the first-order kinetic equation at different temperatures. The selective oxidation reaction rate constants increased significantly with increasing temperature, and the two were positively correlated. Although the time required for the reaction was similar for the four temperature conditions, the difference in reaction rates resulted in different final outputs of glucuronic acid. Specifically, at higher temperatures, molecular motion is intensified, the collision frequency is elevated, and more cellulose molecular chains are involved in the oxidation reaction [24], thereby increasing the selective oxidation efficiency, resulting in more intense oxidation of industrial hemp staple fibers. In particular, the reaction rate constant at 30 °C was 2.2 times higher than that at 0 °C, a finding that highlights the critical role of temperature in modulating oxidation reactions.

According to the Arrhenius Equation: $k=Ae^{-Ea/RT}$ taking logarithmic values yields $\ln k=-\frac{E_{a}}{RT}+\ln A$. The Arrhenius equation was plotted and fitted using the data for temperature and reaction rate constants from Table 2. It is shown in Figure 7c. From Figure 7c, there is a good linear relationship between the different temperatures and the reaction rate constants. The slope after linear regression was −2.70, from which the apparent activation energy was calculated as 22.45 kJ/mol.

### 3.6. SEM Analysis

**Figure 8:**
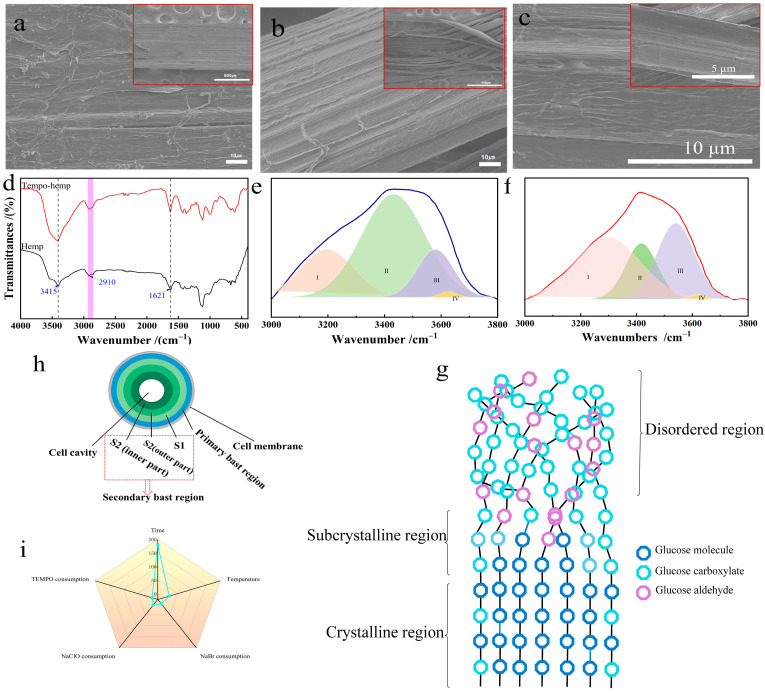
Characterization of properties and model plots. SEM plots ((**a**). industrial hemp staple fiber; (**b**). industrial hemp staple fiber after oxidation by the optimum process; (**c**). industrial hemp staple fiber under similar conditions without oxidizing agents); (**d**). FTIR plots; Gaussian peak fitting and sub-peak distributions at 3000–3800 cm^−1^ ((**e**). industrial hemp staple fiber; (**f**). industrial hemp staple fiber after oxidation by the optimum process); (**g**). microfilament models. (**h**). Schematic diagram of the spatial structure of hemp fiber microfibrils. (**i**). Effect of the conversion rate by factors.

The scanning electron microscope of untreated industrial hemp staple fiber is shown in Figure 8a. The fiber surface is coated with substances such as gum, and the surface is rough, and the fiber bundles are closely arranged [25]. As shown in Figure 8b, the surface of the industrial hemp staple fibers oxidized by the TEMPO system became smooth, and some bundles of fibers split into single fibers. Under similar conditions without oxidizing agents in the control experiment (Figure 8c), the fiber surface exhibited a substantial amount of gum. This indicates that the surface cleaning effect is primarily attributable to TEMPO-mediated oxidation reactions. The TEMPO oxidation system not only removes surface impurities, but also causes minor damage to the fiber surface, a phenomenon consistent with findings from related studies on the de-gumming of hemp fibers using TEMPO systems [26].

### 3.7. Chemical Structure and Hydrogen Bonding Analysis

Figure 8d shows the infrared spectra of industrial hemp staple fibers before and after oxidation by the TEMPO system. The industrial hemp staple fibers before and after oxidation of the TEMPO system had broad peaks at 3415 cm^−1^ and narrow peaks at 2910 cm^−1^, which were attributed to O-H and C-H in cellulose [27], respectively. Additionally, 1621 cm^−1^ corresponds to the C=O tensile vibration of carboxylic acid [28]. The absorption peak of oxidized industrial hemp staple fiber is larger than that of untreated industrial hemp staple fiber. The results indicate that the carboxylic acid group forms during the oxidation reaction, with its content increasing. It was proved that the experimental method of measuring the consumption of NaOH (mL/min) to indirectly monitor the production of glucuronic acid (mol/L) and thus the oxidation reaction rate was feasible.

Cellulose, the main component of industrial hemp staple fiber, is a polyhydroxy compound with a large number of hydrogen bonds [29,30]. The spectral bands of the hydrogen bonding peaks in the range of 3000–3800 cm^−1^ were fitted using the Gaussian fitting method [29,31], as shown in Figure 8e,f. IV (cyclic polymers) and I (OH–OH) belong to intramolecular hydrogen bonding, III (OH–ether O) belongs to intermolecular hydrogen bonding, and II (-OH) belongs to the free hydroxyl group [31]. The fit in the figure shows that as the percentage of intramolecular hydrogen bonding decreases (24.02% to 21.21%), the percentage of intermolecular hydrogen bonding increases (75.98% to 78.79%). This is because the oxygen atom brought by the carboxyl group strengthens the hydrogen bond cooperation network between molecules [32]. The results showed that the selective oxidation of the oxidation kinetics of the TEMPO system acted mainly on the surface of primary and secondary bast fiber microfibers.

### 3.8. Degree of Influence of Different Factors

Comparative analysis of the effect of the five dimensions of TEMPO usage, NaBr usage, NaClO usage, reaction time, and reaction temperature on the conversion rate of industrial hemp staple fiber by selective oxidation, and the results of the analysis of the degree of influence of each factor are shown in Figure 8i. Reaction time had the most significant effect on the conversion of oxidized industrial hemp staple fiber in the TEMPO/NaBr/NaClO system, followed by temperature and NaClO usage. In contrast, TEMPO usage and NaBr usage had the least effect on the conversion of oxidized hemp crude fiber, which provides theoretical support for the control of selective oxidation of the conversion of industrial hemp cellulose. The optimum values of usage were 0.21 mmol/L of TEMPO, 3.24 mmol/L of NaBr, 109.26 mmol/L of NaClO, reaction time 180 min, and reaction temperature 20 °C.

### 3.9. Microfiber Oxidation Model

For the study of the oxidation process, we should consider not only the spatial structure of each layer of microfibrils, but also the existence of crystalline and amorphous regions within a single microfibril (Figure 8h). In the process of establishing the oxidation kinetic equation (Section 3.1), it was found that the reaction rate of the TEMPO/NaBr/NaClO system in oxidizing the primary bast zone of industrial hemp staple fiber was 6.5 times higher than that of the secondary bast zone. We believe the reasons for this phenomenon include the following: (1) the close adhesion between the secondary bast region and the wood pith caused the reaction in the interface region to continue slowly; (2) it was caused by the different arrangement of microfibrils in the secondary bast region [18]; and (3) the crystal structure of cellulose on a smaller scale, namely the amorphous region and crystalline region [33,34]. The first two points have been explained in the reaction rate analysis of oxidation kinetics, so we focus on the third point. The disordered region of cellulose accounts for about 1% to 3% of cellulose [33,34]. The disordered arrangement of molecular chains and the loose structure lead to faster reaction speed, and at least one C_6_ aldehyde group is formed on each cellulose chain as an intermediate structure [24]. With the increase in oxidation time, the macromolecular structure of cellulose becomes more relaxed [13,35]. By studying the crystal structure of cellulose after TEMPO oxidation, it was found that selective oxidation occurred not only in the amorphous region but also on the surface of the crystalline region [21,36], which means that both the primary and secondary phloem regions have crystalline regions involved in the reaction. Zhang Qiang et al. investigated the thermal degradation behavior and thermal degradation kinetics of cotton fibers using the TG-DTG technique under the protection of high-purity nitrogen [37]. They found that the degradation of cotton fibers is a process that occurs excessively from the non-crystalline region to the crystalline region. The kinetic mechanism of the main degradation stage is diffusion-controlled, and the degradation rate is mainly affected by the temperature and decomposition rate. To express more clearly the oxidation of the crystalline, subcrystalline, and disordered zones within the microfibrils during the oxidation of industrial hemp staple fibers mediated by the TEMPO/NaBr/NaClO system to describe the distribution of atomic groups in the internal structure of the cellulose, an ideal model was developed as shown in Figure 8g, without taking into account the non-selective oxidative depolymerization behavior of NaClO. Based on the widely accepted literature regarding the microstructure of cellulose microfibrils and the established mechanism of TEMPO-mediated oxidation initiating preferentially in disordered regions [11,38,39], combined with the kinetic results of this study (which reveal a rapid phase corresponding to oxidation in accessible regions and a slow phase reflecting oxidation in recalcitrant regions), we propose a schematic model (Figure 8g) to illustrate the hypothesized progression of TEMPO-mediated oxidation within the microstructure of industrial hemp bast fibers. This model posits that oxidation advances sequentially from amorphous regions and crystal surfaces toward the interior of the crystals, consistent with the observed biphasic kinetic behavior. It is important to note that this model is a conceptual schematic, intended to visually integrate structural knowledge with kinetic behavior, rather than being derived from computational simulations or representing an atomistically precise structural model.

### 3.10. Limitations of the Kinetic Model

The pseudo-first-order kinetic model employed in this study successfully captures the overall trends of hemp fiber oxidation within the TEMPO system. However, its applicability rests on several key assumptions: a large excess of oxidants (NaClO/NaBr) is required to maintain a steady concentration of active species, and the chemical reaction itself is the rate-determining step. It is crucial to note that this is a practical simplified model. The rate constant it reports is an “apparent” value, integrating both the chemical reaction and the diffusion process of reactants within the fiber structure. Consequently, the model is more adept at comparing the effects of different parameters under the specific experimental conditions of this study than at precisely predicting all complex scenarios. Deviations from the model are expected, particularly during the later stages of oxidation when targeting structurally dense secondary fibers, where increased diffusion resistance becomes significant. The underlying mechanisms for certain complex nonlinear phenomena observed (such as reaction inhibition at high TEMPO concentrations) are further explained by supplementary concepts like our proposed ‘variable-speed competition mechanism,’ which fall outside the scope of the fundamental kinetic model itself.

While the pseudo-first-order model provides excellent fits (high R^2^ values) for the primary reaction phase and allows for effective parameter comparison, its limitations must be acknowledged. The model simplifies the complex heterogeneous reality. Diffusional limitations likely become more pronounced in the later stages of oxidation (especially for the secondary bast fibers) and under conditions of high reagent loading, where reagent penetration into the dense fiber microstructure may become rate-influencing.

## 4. Conclusions

This study systematically elucidated the oxidation mechanism and kinetics of industrial hemp fibers mediated by the TEMPO/NaBr/NaClO system, revealing a novel ‘variable-rate competitive mechanism’ between NaClO and TEMPO. This mechanism explains the observed nonlinear kinetic behavior: excess TEMPO (>0.21 mmol/L) inhibits TEMPOH conversion, disrupting the catalytic cycle and suppressing the overall reaction rate. Additionally, a dynamic equilibrium exists between Ocl^−^ and BrO^−^, regulated by Br^−^ concentration. Optimal NaBr usage (3.24 mmol/L) maximizes active oxidant (Obr^−^) generation, while excessive NaBr leads to Br^−^ accumulation, impeding TEMPO conversion and ultimately slowing cellulose oxidation. TEMPOH formation was identified as the rate-limiting step in TEMPO-related reactions, directly governing selective oxidation efficiency. Under optimized conditions (0.21 mmol/L TEMPO, 3.24 mmol/L NaBr, 109.26 mmol/L NaClO, 180 min, 20 °C), the highest selective oxidation conversion was achieved.

Among operational parameters, reaction time and temperature exerted the most significant influence. Extended reaction time ensured complete oxidation of recalcitrant secondary bast fibers (rate constant k_2_ = 0.18 min^−1^), while elevated temperature accelerated molecular motion, increasing reaction rates as evidenced by low apparent activation energy (Ea = 22.45 kJ/mol). Both primary (k_1_ = 1.17 min^−1^) and secondary phloem regions exhibited pseudo-first-order kinetics, with the primary region reacting 6.5× faster due to distinct spatial structures and cellulose microfibril arrangements.

Collectively, this work provides unprecedented insights into the complex oxidation mechanism of lignin-containing hemp fibers mediated by TEMPO. The discovered ‘variable-rate competitive mechanism’ and identification of rate-limiting steps offer a robust theoretical foundation for optimizing the production of high-value hemp cellulose-based materials.

## Figures and Tables

**Table 1:** Rate constants for the oxidation reaction of primary and secondary bast fibers of industrial hemp staple fibers oxidized by the TEMPO system.

	Reaction Rate Constants/min^−1^	R^2^
Primary bast fibers	1.17	0.998
Secondary bast fibers	0.18	0.958

**Table 2:** Oxidation reaction rate constants and determination coefficient R2 of primary bast fibers of oxidized industrial hemp staple fibers at different temperatures.

	0 °C	10 °C	20 °C	30 °C
Reaction rate constants in primary bast fiber/min^−1^	0.53	0.66	1.14	1.18
Adjust R^2^	0.991	0.998	0.996	0.995

## Data Availability

The datasets used and analyzed during the current study are available from the corresponding author on reasonable request.
